# Current crowding–free superconducting nanowire single-photon detectors

**DOI:** 10.1126/sciadv.adt0502

**Published:** 2025-03-28

**Authors:** Stefan Strohauer, Fabian Wietschorke, Christian Schmid, Stefanie Grotowski, Lucio Zugliani, Björn Jonas, Kai Müller, Jonathan J. Finley

**Affiliations:** ^1^ Walter Schottky Institute, Technical University of Munich, 85748 Garching, Germany.; ^2^ TUM School of Natural Sciences, Technical University of Munich, 85748 Garching, Germany.; ^3^ TUM School of Computation, Information and Technology, Technical University of Munich, 80333 Munich, Germany.; ^4^ Munich Center for Quantum Science and Technology (MCQST), 80799 Munich, Germany.

## Abstract

Detecting single photons is essential for applications such as dark matter detection, quantum science and technology, and biomedical imaging. Superconducting nanowire single-photon detectors (SNSPDs) excel in this task due to their near-unity detection efficiency, subhertz dark count rates, and picosecond timing jitter. However, a local increase of current density (current crowding) in the bends of meander-shaped SNSPDs limits these performance metrics. By locally irradiating the SNSPD’s straight segments with helium ions while leaving the bends unirradiated, we realize current crowding–free SNSPDs with simultaneously enhanced sensitivity: After irradiation with 800 ions nm^−2^, locally irradiated SNSPDs showed a relative saturation plateau width of 37%, while fully irradiated SNSPDs reached only 10%. This larger relative plateau width allows operation at lower relative bias currents, thereby reducing the dark count rate while still detecting single photons efficiently. We achieve an internal detection efficiency of 94% with 7 mHz dark count rate near the onset of saturating detection efficiency for a wavelength of 780 nm.

## Teaser

Local He ion irradiation boosts the detection efficiency and reduces the dark count rate of SNSPDs by mitigating current crowding.

## INTRODUCTION

Superconducting nanowire single-photon detectors (SNSPDs) (*1*) are widely used across fields such as quantum science and technology (*2*–*9*), astronomy (*10*), optical communication (*11*, *12*), biology (*13*, *14*), and medicine (*15*). Their ability to detect single photons with near-unity efficiency (*16*, *17*), subhertz dark count rate (*18*), picosecond timing jitter (*19*), and nanosecond reset time (*20*) makes them ideally suited for demanding applications such as photonic quantum computing (*21*–*27*), particle and dark matter detection (*28*–*31*), or infrared fluorescence microscopy for in vivo deep brain imaging (*13*, *14*). One strategy to further enhance detection efficiency, dark count rate, and timing jitter of SNSPDs is to reduce the effect of current crowding in their bends. Current crowding locally increases the current density in the bends, limiting the maximum applicable bias current through the detector and thus limiting also the maximum achievable detection efficiency, dark count rate, and timing jitter (*32*, *33*). In literature, methods to reduce the effect of current crowding consist of optimized bend geometries (*32*–*36*) or increased superconductor thickness in the bends (*37*, *38*). The use of optimized bend geometries is either limited to relatively low-fill factor SNSPDs, which have lower absorption and detection efficiency, or requires a spiral or special meander design with substantial wire length overhead, which is incompatible with dense SNSPD arrays and limits the timing properties of the detector. In contrast, variable thickness SNSPDs successfully reduce current crowding also for highly efficient and compact large fill factor SNSPDs. In this work, we introduce an innovative method not only to obtain current crowding–free SNSPDs of arbitrary geometry and fill factor but also to simultaneously enhance their sensitivity.

## RESULTS

### Current crowding at low temperatures

Current crowding describes a nonuniform distribution of current density in an electrical conductor and has its origin for example in variations in material properties or device geometry. In the context of SNSPDs, current crowding leads to a local increase of the supercurrent density in the 180° bends and at kinks or discontinuities of the SNSPD, as discussed in more detail in the Supplementary Materials. Once this current density exceeds the critical value for the superconductor, the SNSPD switches to the normal conducting state. This maximum current defines the switching current $I_{\text{sw}}$. In other words, current crowding limits $I_{\text{sw}}$ of the SNSPD.

**Fig. 1.**
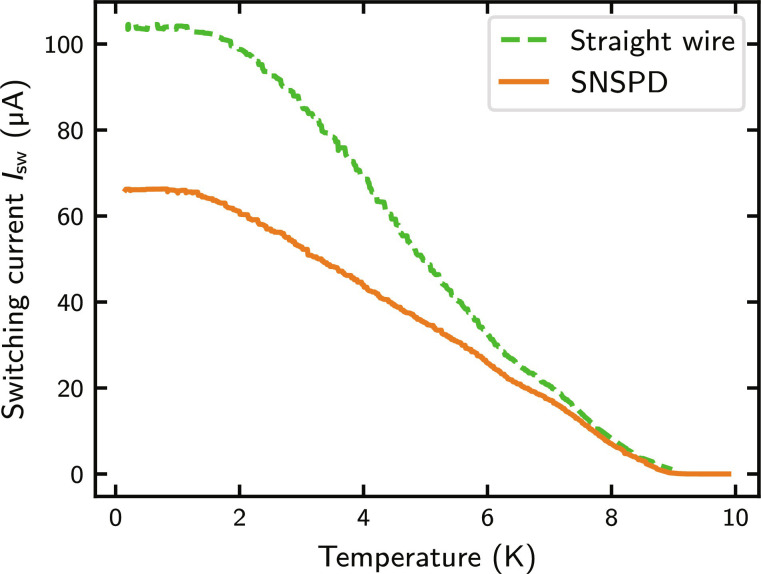
Switching current versus temperature for an SNSPD and a straight wire. Current crowding causes a reduced switching current for the SNSPD over the straight wire, especially toward low temperatures.

It is worthwhile to note that the effect of current crowding is most pronounced at low temperatures ($<0.7\;T_{c}$), while at temperatures close to $T_{c}$, the switching current practically coincides for straight and bent wires due to the divergence of the coherence length at temperatures close to the critical temperature (*39*, *40*). To quantify the effect of current crowding for our devices, we measured the temperature dependence of the switching current for SNSPDs (in meander form with 180° bends) and straight wires (without any bend), both consisting of 250-nm-wide and 8-nm-thick NbTiN wires. As shown for two representative devices in Fig. 1, the switching current increases, and the effect of current crowding becomes more prominent toward low temperatures, reaching a saturating (relative) difference between $I_{\text{sw}}$ of the straight wire and the SNSPD at temperatures below 1.6 K (1 K). At temperatures below 1 K, the switching current of NbTiN meander SNSPDs with a fill factor of 71% and 250-nm-wide wires is reduced to only 60% of $I_{\text{sw}}$ of corresponding straight wires.

At the same time, a high switching current, as close as possible to the limit set by the depairing current density, is desirable because it allows larger plateaus of saturating detection efficiency (*19*, *41*). This makes operation with high detection efficiency at simultaneously low dark count rates possible. Moreover, high switching currents allow high bias currents through the SNSPDs that yield high and easily detectable voltage pulses and low timing jitter (*38*). Because high switching currents are reached for low temperatures where current crowding limits the maximum applicable bias current, a method to avoid current crowding would be highly advantageous. Moreover, current crowding–free SNSPDs are expected to exhibit fewer dark counts because they originate primarily from bends or constrictions with strong current crowding (*34*, *37*, *42*–*44*). Compared to methods presented in literature that solely mitigate current crowding (*32*–*38*), the following section introduces a solution to obtain current crowding–free SNSPDs of arbitrary geometry that simultaneously exhibit enhanced sensitivity.

### Design and concept of current crowding–free SNSPDs with enhanced sensitivity

**Fig. 2.**
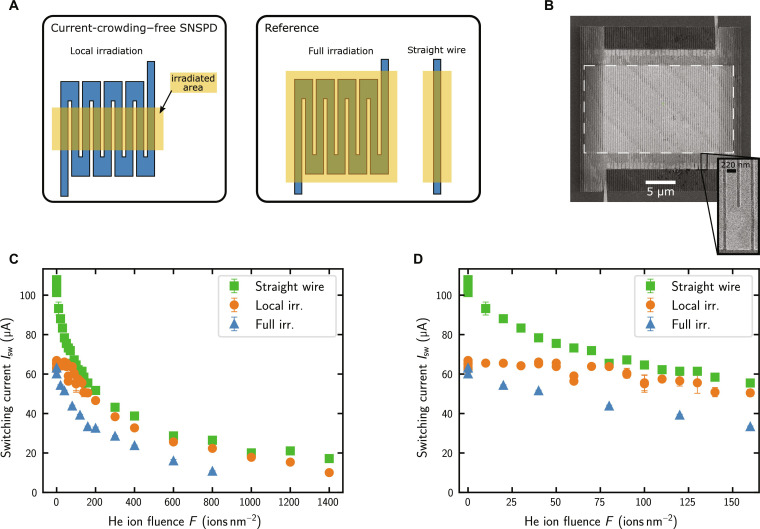
Current crowding–free SNSPDs. (**A**) Local irradiation of the straight detector segments is used to obtain current crowding–free SNSPDs. Fully irradiated SNSPDs and straight wires were fabricated as reference devices. (**B**) Scanning electron microscope image of an SNSPD after local irradiation. The irradiation area is indicated by the white dashed rectangle. (**C** and **D**) Switching current versus He ion fluence for locally and fully irradiated SNSPDs and straight wires for the low fluence regime (C) and for the full measured range up a fluence of 1400 ions nm^−2^ (D).

To demonstrate current crowding–free SNSPDs of enhanced sensitivity, we combine the two effects of reduced switching current and enhanced sensitivity of SNSPDs after helium (He) ion irradiation (*45*, *46*). As discussed in the previous section, the 180° bends are the main contribution to current crowding in a meander-type SNSPD, thus limiting its switching current. By locally irradiating only the straight segments of the SNSPD while leaving its bends unirradiated, we reduce the critical current density of the straight segments while making them simultaneously more sensitive to single photons. Once the He ion fluence is large enough, the critical current of the straight segments with homogeneous current density will be lower than the critical current of the bends. Then, one can expect the overall critical current (or switching current) of the SNSPD to be given by the critical current of the straight segments and not being limited by current crowding anymore. To investigate this hypothesis, three types of devices as shown in Fig. 2A were fabricated on the same chip: SNSPDs that were locally (only their straight segments) or fully irradiated as well as straight wires without any bend. They were fabricated from an 8-nm-thick NbTiN film on a Si substrate with 130-nm thermally grown SiO_2_. The nominal design of the detectors consists of 250-nm-wide nanowires in meander form with 100-nm gaps (fill factor 71%) and covers an active area of 20 μm by 20 μm. This high fill factor was chosen to have a pronounced effect of current crowding. Simulations for this geometry are provided in the Supplementary Materials and demonstrate that it is strongly affected by current crowding. The reference straight wires were fabricated on the same chip, also with a wire width of 250 nm. All devices were characterized before irradiation by measuring their switching current distributions and mean switching currents at a temperature of 1 K. After characterization of the unirradiated devices, a He ion microscope (Zeiss Orion Nanofab) with an acceleration voltage of 30 kV was used to irradiate them with He ions. An irradiation area of 25 μm by 15 μm was centered on the 20 μm by 20 μm detectors to homogeneously expose the straight segments of the nanowires while leaving the bends unirradiated. In this way, locally irradiated SNSPDs as shown in Fig. 2 (A and B) were obtained. For the reference straight wires, an irradiation area of 5 μm by 15 μm was chosen. Moreover, two detectors were fully irradiated (irradiation area of 25 μm by 25 μm). After irradiation, the devices were characterized again, and for several devices, the process of irradiation and subsequent measurement was repeated multiple times.

### Reducing current crowding by local He ion irradiation

As shown in Fig. 2 (C and D), the mean switching current of locally irradiated SNSPDs stays constant for doses up to 90 ± 20 ions nm^−2^, while it continuously decreases for fully irradiated SNSPDs and straight wires. As anticipated, locally irradiated SNSPDs exhibit a constant $I_{\text{sw}}$ until the He ion fluence reaches a level that reduces $I_{\text{sw}}$ of the straight wires to comparable values. Beyond this point, $I_{\text{sw}}$ of locally irradiated SNSPDs and straight wires follow a similar curve. The slightly smaller $I_{\text{sw}}$ of locally irradiated SNSPDs compared to that of the straight wires can be attributed to the higher likelihood of imperfections in one of the many straight segments of an SNSPD, unlike in a single straight wire.

**Fig. 3.**
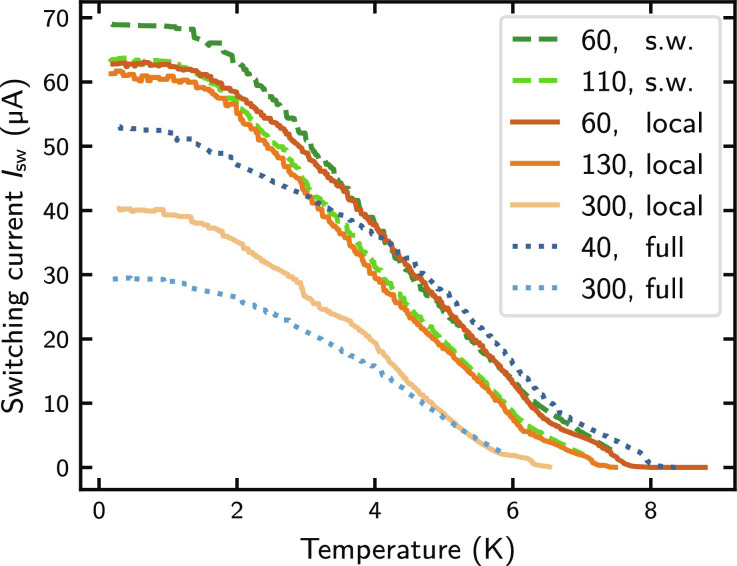
Switching current of straight wires (s.w.), locally irradiated SNSPDs (local), and fully irradiated SNSPDs (full) versus temperature. The He ion fluence used for irradiation is given by the numbers in front of the device type in units of ions nm^−2^.

Figure 3 shows the temperature dependence of the switching current for differently irradiated SNSPDs and straight wires. The data shown are the maximum of the switching current distribution measured at each temperature (*40*, *47*). The critical temperature decreases continuously with increasing He ion fluence in accordance with literature (*45*, *46*) and does not depend on device type or irradiation type. For decreasing temperature, the $I_{\text{sw}}\left(T\right)$ curves start to differ and show a smaller slope for fully irradiated SNSPDs compared to that of locally irradiated SNSPDs and irradiated straight wires. Moreover, for similar He ion fluences and low temperatures, $I_{\text{sw}}$ is much smaller for fully irradiated SNSPDs than for locally irradiated SNSPDs or straight wires. For example, at temperatures below 1 K and for a He ion fluence of 300 ions nm^−2^, it is only 30 μA instead of 40 μA. While for a He ion fluence of 60 ions nm^−2^, the switching current of the straight wire is still higher than that of the locally irradiated SNSPD, and they almost coincide for the two fluences 110 and 130 ions nm^−2^. Those observations support the hypothesis that for small He ion fluences, $I_{\text{sw}}$ of the locally irradiated SNSPDs is not reduced, while it starts to coincide with that of the straight wires for fluences above 90 ± 20 ions nm^−2^. In this case, the irradiation induced reduction of the switching current in the straight segments is the limiting factor for $I_{\text{sw}}$ instead of current crowding in the bends. The stronger the effect of current crowding in the unirradiated device, the higher the He ion fluence is required for local irradiation such that current crowding no longer plays a role. Because current crowding limits the switching current of SNSPDs more as the fill factor increases, this method is particularly useful for high fill factor or microscale SNSPDs, especially if the detector requires He ion irradiation anyway to become single-photon sensitive (*48*, *49*).

### Enhanced sensitivity of current crowding–free SNSPDs

**Fig. 4.**
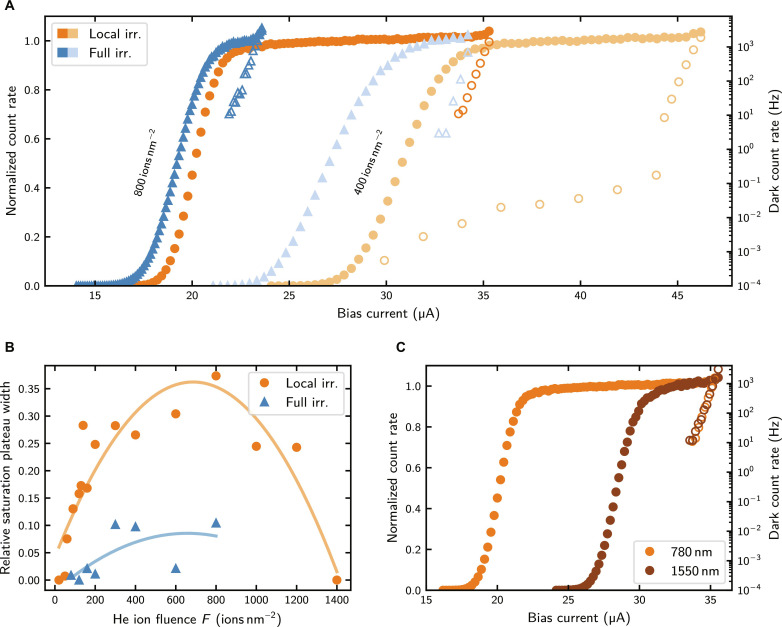
Detection performance of SNSPDs after local or full irradiation with He ions. (**A**) Normalized count rate (solid symbols) and dark count rate (open symbols) versus bias current after local (orange) or full (blue) irradiation with He ions. The He ion fluence used for irradiation is shown as annotations at the corresponding count rate curves. (**B**) Relative saturation plateau width versus He ion fluence after local or full irradiation, calculated according to Eq. 1. The solid lines serve as a guide to the eye. (**C**) Comparison of the normalized count rate between photons of 780- and 1550-nm wavelength for a detector locally irradiated with 800 ions nm^−2^. All shown dark count rate data were taken such that the integration time per data point was at least 10 times higher than the inverse of the measured dark count rate. All data shown were measured at a temperature of 1 K using photons with a wavelength of 780 nm, unless explicitly stated otherwise.

Helium ion irradiation enhances the sensitivity of SNSPDs. However, because we do not irradiate the whole SNSPD but intentionally leave the bends unirradiated and, consequently, do not have the irradiation induced decrease of switching current, we expect an enhanced performance due to two effects: (i) the He ion irradiation induced higher sensitivity and (ii) the higher available bias currents compared to fully irradiated SNSPDs of the same He ion fluence. Figure 4A shows a comparison of the normalized count rates between two devices, one irradiated locally and one irradiated fully, both with the same He ion fluences. All data in Figure 4 were measured for photons of 780-nm wavelength and at a temperature of 1 K, unless explicitly stated otherwise. The locally irradiated SNSPD shows higher switching currents and larger saturation plateaus of the count rates, for which the internal detection efficiency is unity. We note that, rather than a flat saturation plateau, the normalized count rate for the locally irradiated SNSPD shows a continuous, slight increase with increasing bias current. Because of scattering and recoil events of the He ions within the sample (*50*), we expect a gradual rather than abrupt decrease in sensitivity at the edges of the area scanned with the He ion microscope during irradiation. When the bias current is sufficiently high for the central region with homogeneous sensitivity to reach a saturating count rate, the edges of the irradiated area remain below saturation. As the bias current increases further, the count rate in these edge regions continues to increase, resulting in the observed slight increase in the overall count rate. The dark count rate at a certain bias current $I_{b}$ mainly depends exponentially on the ratio $I_{b}/I_{\text{sw}}$. Therefore, the dark count rate of locally irradiated SNSPDs is lower than that of fully irradiated SNSPDs at similar absolute bias currents or normalized count rates. Because our SNSPDs exhibit very low dark count rates, we measured it over an extended period of time for selected devices. As shown in Fig. 4A for the device locally irradiated with 400 ions nm^−2^, we reach dark count rates of 7 and 0.55 mHz at internal detection efficiencies of 94 and 31%, respectively. Also, the two regimes of intrinsic dark counts and dark counts originating from black body radiation are visible: The first is given by the strongly current dependent contribution in the high current regime, while the second is the weaker current-dependent contribution at lower currents (*51*). The black body radiation induced dark count rate could be further suppressed by using cold bandpass filters (*52*).

In Fig. 4B, we present the relative count rate saturation plateau width, defined as

$$\sigma_{\text{rel}}=\frac{I_{c}-I_{\text{sat}}}{I_{c}}$$ (1)

with the critical current $I_{c}$ and $I_{\text{sat}}$ representing the current where the saturation plateau begins (where the normalized count rate reaches 0.95). The critical current is defined as the smallest bias current where the SNSPD shows nonzero resistance. Because we use a shunt resistor for the count rate measurements to prevent latching of the detectors, the SNSPDs transition to the relaxation oscillation regime at $I_{c}$ before switching to the latching state (*53*). The relative saturation plateau width is higher for locally irradiated SNSPDs compared to fully irradiated SNSPDs of the same He ion fluence. Furthermore, the relative saturation plateau width increases strongest as a function of He ion fluence for values smaller than 200 ions nm^−2^ and, as elaborated in the Supplementary Materials, it peaks between 600 and 1000 ions nm^−2^ before it decreases again for higher fluences. To assess the performance of the device locally irradiated with 800 ions nm^−2^ for optical communication in the C-band, we measured its normalized count rate for photons with a wavelength of 1550 nm and compare it in Fig. 4C with its performance for 780-nm photons. For both wavelengths, the detector shows saturating internal detection efficiency, even for 1550 nm where the onset of photon detection is shifted to higher bias currents due to the lower sensitivity to longer wavelength photons. Because it is the same device, the dark count rate is identical for both measurements.

Moreover, we found that the detection pulse height (relevant for assessing the requirements of the readout electronics) of locally irradiated SNSPDs is higher than that of fully irradiated SNSPDs of the same fluence, while the pulse decay time (related to the detector’s dead time) is similar. These quantities are further discussed in the Supplementary Materials.

## DISCUSSION

Current crowding strongly limits the maximum bias current, sensitivity, and dark count rate of SNSPDs at low temperatures. Therefore, reducing the effect of current crowding is crucial for applications that require low dark count rates and high detection efficiency. We demonstrated a method to obtain current crowding–free SNSPDs by irradiating the straight segments of SNSPDs locally with He ions while leaving their bends unirradiated. Up to a He ion fluence of 90 ± 20 ions nm^−2^, the critical current is not reduced, meaning the photon sensitivity is enhanced without sacrificing any of the SNSPD‘s critical current. Above this threshold, the straight segments of the locally irradiated SNSPDs show a critical current lower than that of the bends such that current crowding in the bends does not limit the overall critical current anymore. The boost in performance for locally irradiated SNSPDs is twofold: (i) The sensivitiy of the SNSPD is enhanced due to He ion irradiation, and (ii) the sensitivity is higher than for fully irradiated SNSPDs due to the higher available bias currents. Using this approach, we achieved a relative saturation plateau width of 37% for a locally irradiated SNSPD compared to 10% for a fully irradiated SNSPD, both irradiated with 800 ions nm^−2^. This larger relative plateau width means that the SNSPD can be operated at lower relative bias currents with lower dark count rates and still detect single photons efficiently (e. g., 7 mHz dark count rate at an internal detection efficiency of 94%). Therefore, this method is particularly beneficial for applications that require high detection efficiency combined with low dark count rate, such as quantum key distribution, brain imaging, and dark matter detection (*7*, *13*, *30*). Furthermore, it is ideally suited for high fill factor or micro-scale SNSPDs that are prone to current crowding, especially if the detectors require He ion irradiation anyway to become single-photon sensitive (*48*). Compared to methods that avoid current crowding by special meander designs (*32*–*36*) or by variable thickness SNSPDs (*37*, *38*), this solution not only yields current crowding–free SNSPDs of arbitrary geometry and fill factor but also simultaneously enhances their sensitivity. By combining site selective (*46*) with local He ion irradiation, one can realize highly and homogeneously performing SNSPD arrays. Moreover, local He ion irradiation can be further used to study the effect of current crowding in different regions of superconducting devices, including SNSPDs and the influence of current crowding in their bends onto properties such as dark count rate, detection efficiency, and switching current.

## MATERIALS AND METHODS

### Fabrication of NbTiN SNSPDs

To fabricate SNSPDs and straight wires, we deposited an 8-nm-thick NbTiN film using DC reactive magnetron sputtering onto a Si substrate with a 130-nm-thick thermally grown SiO_2_ layer. The superconductor thickness was controlled by measuring the sputtering rate and choosing the sputtering time correspondingly. Subsequently, we patterned the SNSPDs and straight wires with electron beam lithography and reactive ion etching, followed by contact pad fabrication using optical lithography and gold evaporation (*54*). The detector design consists of 250-nm-wide nanowires in meander form with 100-nm gaps (fill factor of 71%) and covers an active area of 20 μm by 20 μm. The high fill factor of 71% and a nominally rectangular design of the bends between neighboring nanowires of the SNSPDs were chosen to have a pronounced effect of current crowding as elaborated in more detail in the Supplementary Materials. However, because of fabrication limitations, the actual bends have a relatively circular shape as shown in the zoom-in of Fig. 2B. Similar to the detectors, the straight wires were designed to be 250-nm wide and fabricated on the same chip to ensure best comparability. After characterization of the unirradiated devices, we used a He ion microscope (Zeiss Orion Nanofab) for irradiation with He ions with an acceleration voltage of 30 kV. For the locally irradiated devices, we chose an irradiation area of 25 μm by 15 μm, centered on the 20 μm–by–20 μm detectors. Two detectors were fully irradiated with an irradiation area of 25 μm by 25 μm. For the reference straight wires, an irradiation area of 5 μm by 15 μm was chosen. After irradiation, we characterized the devices again and repeated the process of irradiation and subsequent measurements for several devices multiple times.

### Low-temperature measurements

All detectors were precharacterized in a cryogenic probe station (Janis) at 4.5 K. Subsequently, devices exhibiting a similar switching current of 42 ± 2 μA at 4.5 K were selected for wire bonding and further characterization at temperatures down to 150 mK in a closed cycle cryostat with adiabatic demagnetization refrigeration (kiutra GmbH). Further characterization consisted of measuring the switching current of each device 10 to 250 times to determine a switching current distribution and its mean switching current at a temperature of 1 K. The switching current measurements were performed by ramping the bias voltage applied at a 50 kilohm resistor in series to the respective device and determining the current at which the total resistance increases by more than 1 kilohm. Temperature-dependent switching current measurements were performed at temperature ramp rates of 0.075 K/min, ensuring that during one bias voltage sweep, the temperature change was less than 0.1 K.

## Supplementary Materials

### This PDF file includes:

Supplementary Text Figs. S1 to S4 References
