# Supplementary material for: Current crowding–free superconducting nanowire single-photon detectors
Source: Sci Adv. 2025 Mar 28;11(13):eadt0502. doi: 10.1126/sciadv.adt0502 (PMC11952108; doi:10.1126/sciadv.adt0502)
Supplement: Supplementary file 1 — Supplementary Text Figs. S1 to S4 References [file sciadv.adt0502_sm.pdf]

Science Advances
AAAS

Supplementary Materials for

Current crowding–free superconducting nanowire single-photon detectors

Stefan Strohauer *et al.*

Corresponding author: Stefan Strohauer, stefan.strohauer@tum.de; Jonathan J. Finley, jj.finley@tum.de

Sci. Adv. **11**, eadt0502 (2025)
DOI: 10.1126/sciadv.adt0502

This PDF file includes:

Supplementary Text
Figs. S1 to S4
References

Supplementary Text

Simulation of current crowding in SNSPD bends

As detailed in Section 2.1, current crowding in the 180° bends and at kinks/discontinuities of SNSPDs substantially reduces the switching current of these devices, particularly at low temperatures. Since the shape of the 180° bends influences the severity of current crowding (*32*), this section presents simulations how the ellipticity ϵ of the bends impacts the current distribution. Using a methodology similar to that described in the supporting information of (*55*), we perform simulations using the Comsol Multiphysics software. We start from the fact that the supercurrent density fulfills the continuity equation for a steady current, $\nabla \cdot \mathbf{J} = 0$, and the London equation, $\nabla \times \mathbf{J} = 0$, in which the effects of the magnetic field are neglected (*32, 55*). The supercurrent density can be described as the gradient $\mathbf{J} = \nabla u$ of a scalar potential u, since this specific formulation is generally valid when the current density satisfies the London equation ($\nabla \times (\nabla u) = 0$). Combining the definition $\mathbf{J} = \nabla u$ with the continuity equation, the scalar potential satisfies the Laplace equation $\Delta u = 0$. Since the electrostatics module of Comsol solves the Laplace equation for the electric potential, we use this module to calculate the supercurrent density $\mathbf{J}$. As boundary conditions we choose the gradient of the electric potential to match the bias current density at the inflow boundary (one end facet of the wire), $\nabla u = \mathbf{J}_{\mathrm{bias}}$, and set $u = 0$ at the outflow boundary (the other end facet of the wire). For all other boundaries we set $\nabla u = 0$ to ensure no inward or outward current flow.

For our simulations, we define the ellipticity of the 180° bends of the SNSPD as $\epsilon = 1 - b/a$, with the semi-axis a being the vertical gap distance between the wires, and the semi-axis b defining the horizontal elongation of the bend. Moreover, we choose wire widths between 190 nm and 250 nm with a fixed pitch of 350 nm. While the detectors used in this work have a designed wire width of 250 nm, SEM measurements reveal an actual wire width of 220 ± 10 nm, as shown in the zoom-in of Figure 2B. Figure S1 shows the ratio of the homogeneous current density in the straight parts J_{straight} to the maximum current density of the whole bend J_{max}. Especially as the bends approach a rectangular shape ($\epsilon \rightarrow 1$), these simulations show that current crowding drastically reduces this ratio. The gray shaded area indicates the range between 58 % and 66 % to which the experimentally measured switching current of our SNSPDs is reduced compared to that of the straight wires. The three exemplary bends of different ellipticity in Figure S1 visualize the distribution of the

normalized current density in the bend and show a maximum current density in the strongly curved regions. Despite the fact that our detector design consists of rectangular bends in order to have a pronounced effect of current crowding for this study, SEM images such as the zoom-in of Figure 2B show that due to fabrication limitations, the actual bends have a relatively circular shape ($\epsilon \approx 0$). Comparing the corresponding simulation results with the experimentally measured reduction in switching current due to current crowding, we conclude that the experimental reduction is slightly smaller than expected. However, the effective wire width may be smaller than the width measured with the SEM due to oxidation (*56*) and fabrication limitations. Moreover, considering more streamline-shaped bends (*32*), the simulated ratio $J_{\mathrm{straight}}/J_{\mathrm{max}}$ would be a bit higher than in our simulations of elliptical bends. Both effects would result in a larger overlap between experiment and simulation. Furthermore, we conclude that SNSPDs with higher fill factors or sharper, more rectangular bends than those studied in our experiments would experience an even greater reduction in switching current due to increased current crowding. Such devices would particularly benefit from the irradiation scheme proposed in this work.

Detection pulse characterization

In this section, we analyze the recovery time and pulse height of a detection pulse after photon absorption, since both are important performance parameters of single-photon detectors.

The recovery time determines the detector's maximum count rate and can be estimated from the time constant τ_{d} of the exponential decay of a detection voltage pulse (*23, 57*). As shown in Figure S2, the decay time of all device types increases with increasing He ion fluence in agreement with previous measurements (*46*). At the same time, the decay time of locally irradiated SNSPDs follows the same curve as that of fully irradiated SNSPDs, while pulses of straight wires show a significantly faster decay due their smaller kinetic inductance. Thus, local irradiation shows no drawback with respect to the decay time compared to full irradiation of SNSPDs.

As shown in Figure S3 the pulse height of locally irradiated SNSPDs is higher than that of fully irradiated SNSPDs due to higher available bias currents after irradiating only locally. This is beneficial since higher detection voltage pulses are easier to process for the readout electronics.

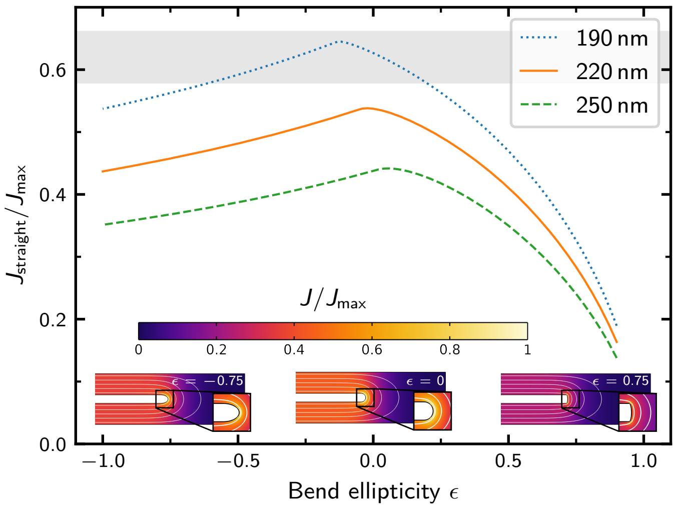

Figure S1: **Simulated current-crowding-induced reduction of the switching current in SNSPDs versus the ellipticity ϵ of the bends.** The reduction is given by the ratio of the homogeneous current density in the straight parts and the maximum current density of the whole bend (located in the strongly curved regions). This ratio is calculated for three different wire widths ranging from 190 nm to 250 nm and a fixed pitch of 350 nm. The gray shaded area indicates the range between 58 % and 66 % to which the experimentally measured switching current of our SNSPDs is reduced compared to that of the straight wires. The three exemplary bend structures show the current density distribution throughout the bends for a wire width of 250 nm and different ellipticities.

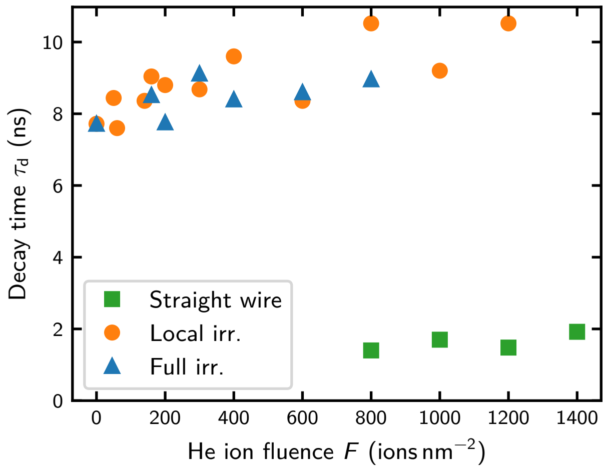

Figure S2: **Decay time versus He ion fluence.** The data shows locally irradiated SNSPDs together with fully irradiated SNSPDs and straight wires as reference devices.

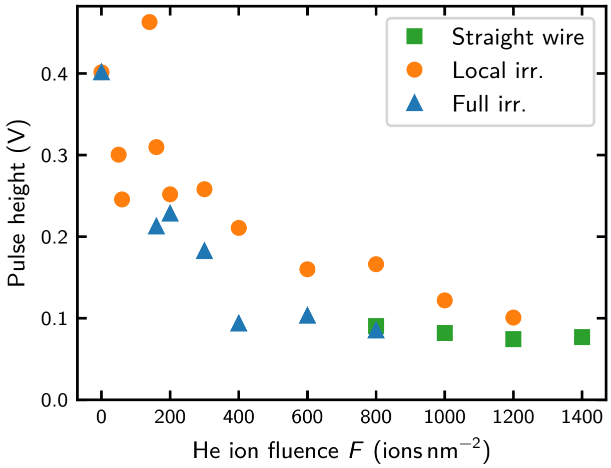

Figure S3: **Pulse height versus He ion fluence.** The data shows locally irradiated SNSPDs together with fully irradiated SNSPDs and straight wires as reference devices.

Saturation plateau width of locally irradiated SNSPDs

The relative saturation plateau width is given by

$$\sigma_{\mathrm{rel}} = \frac{I_{\mathrm{c}} - I_{\mathrm{sat}}}{I_{\mathrm{c}}} \,, \tag{S1}$$

with the absolute saturation plateau size given by the difference between the critical current I_{c} and the current I_{sat} where the saturation plateau begins. Since we use a shunt resistor for the CR measurements to prevent latching of the detectors (20.4 Ω at 1 K), the SNSPDs transition to the relaxation oscillation regime at I_{c} before switching to the latching state. In this regime the SNSPD emits a periodic train of voltage pulses and the average voltage drop across the SNSPD increases with increasing bias current (*53*). Figure S4A shows the absolute saturation plateau width $I_{\mathrm{c}} - I_{\mathrm{sat}}$ where a steep increase in saturation plateau width is observed for He ion fluences up to 200 ions nm^{-2}, followed by a decrease beyond 600 ions nm^{-2}. At the same time, the critical current decreases with increasing He ion fluence. As shown in Figure S4B, the resulting relative saturation plateau width increases for small He ion fluences, peaks between 600 ions nm^{-2} and 1000 ions nm^{-2} before it decreases again for higher fluences. This relation is even better visible in Figure S4C after normalizing the saturation plateau width to the switching current of the devices without shunt resistor instead of the critical current during CR measurements.

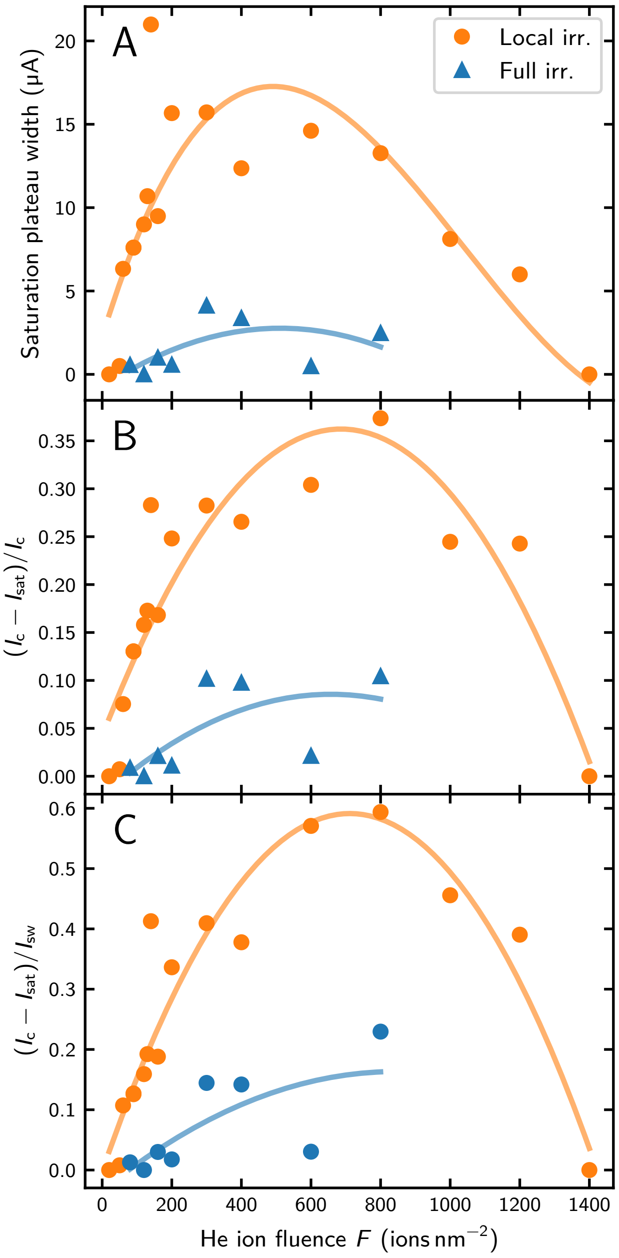

Figure S4: **Count rate saturation plateau width of locally and fully irradiated SNSPDs versus He ion fluence.** (**A**) Absolute saturation plateau width. (**B**) Relative saturation plateau width as given in Equation (S1). (**C**) Saturation plateau width, normalized to the switching current of the corresponding devices measured without shunt resistor. The solid lines serve as a guide to the eye.

REFERENCES AND NOTES

1. G. N. Gol'tsman, O. Okunev, G. Chulkova, A. Lipatov, A. Semenov, K. Smirnov, B. Voronov, A. Dzardanov, C. Williams, R. Sobolewski, Picosecond Superconducting single-photon optical detector. *Appl. Phys. Lett.* **79**, 705–707 (2001).

2. H. Takesue, S. W. Nam, Q. Zhang, R. H. Hadfield, T. Honjo, K. Tamaki, Y. Yamamoto, Quantum key distribution over a 40-dB Channel loss using superconducting single-photon detectors. *Nat. Photonics* **1**, 343–348 (2007).

3. J.-P. Chen, C. Zhang, Y. Liu, C. Jiang, D.-F. Zhao, W.-J. Zhang, F.-X. Chen, H. Li, L.-X. You, Z. Wang, Y. Chen, X.-B. Wang, Q. Zhang, J.-W. Pan, Quantum key distribution over 658 km fiber with distributed vibration sensing. *Phys. Rev. Lett.* **128**, 180502 (2022).

4. Y. Liu, W.-J. Zhang, C. Jiang, J.-P. Chen, C. Zhang, W.-X. Pan, D. Ma, H. Dong, J.-M. Xiong, C.-J. Zhang, H. Li, R.-C. Wang, J. Wu, T.-Y. Chen, L. You, X.-B. Wang, Q. Zhang, J.-W. Pan, Experimental twin-field quantum key distribution over 1000 km fiber distance. *Phys. Rev. Lett.* **130**, 210801 (2023).

5. F. Bussières, C. Clausen, A. Tiranov, B. Korzh, V. B. Verma, S. W. Nam, F. Marsili, A. Ferrier, P. Goldner, H. Herrmann, C. Silberhorn, W. Sohler, M. Afzelius, N. Gisin, Quantum teleportation from a telecom-wavelength photon to a solid-state quantum memory. *Nat. Photonics* **8**, 775–778 (2014).

6. H. Takesue, S. D. Dyer, M. J. Stevens, V. Verma, R. P. Mirin, S. W. Nam, Quantum teleportation over 100 km of fiber using highly efficient superconducting nanowire single-photon detectors. *Optica* **2**, 832 (2015).

7. H. Shibata, T. Honjo, K. Shimizu, Quantum key distribution over a 72 dB channel loss using ultralow dark count superconducting single-photon detectors. *Opt. Lett.* **39**, 5078–5081 (2014).

8. R. Valivarthi, M. G. Puigibert, Q. Zhou, G. H. Aguilar, V. B. Verma, F. Marsili, M. D. Shaw, S. W. Nam, D. Oblak, W. Tittel, Quantum teleportation across a metropolitan fibre network. *Nat. Photonics* **10**, 676–680 (2016).

9. C. M. Natarajan, M. G. Tanner, R. H. Hadfield, Superconducting nanowire single-photon detectors: Physics and applications. *Supercond. Sci. Technol.* **25**, 063001 (2012).

10. E. E. Wollman, V. B. Verma, A. B. Walter, J. Chiles, B. Korzh, J. P. Allmaras, Y. Zhai, A. E. Lita, A. N. McCaughan, E. Schmidt, S. Frasca, R. P. Mirin, S. W. Nam, M. D. Shaw, Recent advances in superconducting nanowire single-photon detector technology for exoplanet transit spectroscopy in the mid-infrared. *J. Astron. Telesc. Instrum. Syst.* **7**, 011004 (2021).

11. M. E. Grein, A. J. Kerman, E. A. Dauler, M. M. Willis, B. Romkey, R. J. Molnar, B. S. Robinson, D. V. Murphy, D. M. Boroson, An Optical Receiver for the Lunar Laser Communication Demonstration Based on Photon-Counting Superconducting Nanowires (*Advanced Photon Counting Techniques IX*, 2015), vol. 9492, pp. 11–16); doi:10.1117/12.2179781.

12. A. Biswas, M. Srinivasan, R. Rogalin, S. Piazzolla, J. Liu, B. Schratz, A. Wong, E. Alerstam, M. Wright, W. T. Roberts, J. Kovalik, G. Ortiz, A. Na-Nakornpanom, M. Shaw, C. Okino, K. Andrews, M. Peng, D. Orozco, W. Klipstein, Status of NASA's deep space optical communication technology demonstration, in *2017 IEEE International Conference on Space Optical Systems and Applications (ICSOS)* (IEEE, 2017), pp. 23–27; doi:10.1109/ICSOS.2017.8357206.

13. F. Xia, M. Gevers, A. Fognini, A. T. Mok, B. Li, N. Akbari, I. E. Zadeh, J. Qin-Dregely, C. Xu, Short-wave infrared confocal fluorescence imaging of deep mouse brain with a superconducting nanowire single-photon detector. *ACS Photonics* **8**, 2800–2810 (2021).

14. A. Tamimi, M. Caldarola, S. Hambura, J. C. Boffi, N. Noordzij, J. W. N. Los, A. Guardiani, H. Kooiman, L. Wang, C. Kieser, F. Braun, A. Fognini, R. Prevedel, Deep mouse brain two-photon near-infrared fluorescence imaging using a superconducting nanowire single-photon detector array. *ACS Photonics* **11**, 3960–3971 (2024).

15. N. Ozana, A. I. Zavriyev, D. Mazumder, M. B. Robinson, K. Kaya, M. H. Blackwell, S. A. Carp, M. A. Franceschini, Superconducting nanowire single-photon sensing of cerebral blood flow. *Neurophotonics* **8**, 035006 (2021).

16. F. Marsili, F. Bellei, F. Najafi, A. E. Dane, E. A. Dauler, R. J. Molnar, K. K. Berggren, Efficient single photon detection from 500nm to 5μm wavelength. *Nano Lett.* **12**, 4799–4804 (2012).

17. A. Korneev, Y. Korneeva, I. Florya, B. Voronov, G. Goltsman, NbN nanowire superconducting single-photon detector for mid-infrared. *Phys. Procedia* **36**, 72–76 (2012).

18. H. Shibata, K. Shimizu, H. Takesue, Y. Tokura, Ultimate low system dark-count rate for superconducting nanowire single-photon detector. *Opt. Lett.* **40**, 3428–3431 (2015).

19. B. Korzh, Q. Y. Zhao, J. P. Allmaras, S. Frasca, T. M. Autry, E. A. Bersin, A. D. Beyer, R. M. Briggs, B. Bumble, M. Colangelo, G. M. Crouch, A. E. Dane, T. Gerrits, A. E. Lita, F. Marsili, G. Moody, C. Peña, E. Ramirez, J. D. Rezac, N. Sinclair, M. J. Stevens, A. E. Velasco, V. B. Verma, E. E. Wollman, S. Xie, D. Zhu, P. D. Hale, M. Spiropulu, K. L. Silverman, R. P. Mirin, S. W. Nam, A. G. Kozorezov, M. D. Shaw, K. K. Berggren, Demonstration of Sub-3 Ps temporal resolution with a superconducting nanowire single-photon detector. *Nat. Photonics* **14**, 250–255 (2020).

20. S. Cherednichenko, N. Acharya, E. Novoselov, V. Drakinskiy, Low kinetic inductance superconducting MgB_2 nanowires with a 130 ps relaxation time for single-Photon detection applications. *Supercond. Sci. Technol.* **34**, 044001 (2021).

21. S. Slussarenko, G. J. Pryde, Photonic quantum information processing: A concise review. *Appl. Phys. Rev.* **6**, 041303 (2019).

22. S. Gyger, J. Zichi, L. Schweickert, A. W. Elshaari, S. Steinhauer, S. F. Covre Da Silva, A. Rastelli, V. Zwiller, K. D. Jöns, C. Errando-Herranz, reconfigurable photonics with on-chip single-photon detectors. *Nat. Commun.* **12**, 1408 (2021).

23. S. Ferrari, C. Schuck, W. Pernice, Waveguide-integrated superconducting nanowire single-photon detectors. *Nanophotonics* **7**, 1725–1758 (2018).

24. J. P. Sprengers, A. Gaggero, D. Sahin, S. Jahanmirinejad, G. Frucci, F. Mattioli, R. Leoni, J. Beetz, M. Lermer, M. Kamp, S. Höfling, R. Sanjines, A. Fiore, Waveguide superconducting

single-photon detectors for integrated quantum photonic circuits. *Appl. Phys. Lett.* **99**, 181110 (2011).

25. G. Reithmaier, S. Lichtmannecker, T. Reichert, P. Hasch, K. Müller, M. Bichler, R. Gross, J. J. Finley, On-chip time resolved detection of quantum dot emission using integrated superconducting single photon detectors. *Sci. Rep.* **3**, 1901 (2013).

26. G. Reithmaier, M. Kaniber, F. Flassig, S. Lichtmannecker, K. Müller, A. Andrejew, J. Vučković, R. Gross, J. J. Finley, On-chip generation, routing, and detection of resonance fluorescence. *Nano Lett.* **15**, 5208–5213 (2015).

27. S. Majety, S. Strohauer, P. Saha, F. Wietschorke, J. J. Finley, K. Müller, M. Radulaski, Triangular quantum photonic devices with integrated detectors in silicon carbide. *Mater. Quantum. Technol.* **3**, 015004 (2023).

28. T. Polakovic, W. Armstrong, G. Karapetrov, Z. E. Meziani, V. Novosad, Unconventional applications of superconducting nanowire single photon detectors. *Nanomaterials* **10**, 1198 (2020).

29. M. Shigefuji, A. Osada, M. Yabuno, S. Miki, H. Terai, A. Noguchi, Efficient low-energy single-electron detection using a large-area superconducting microstrip. arXiv:2301.11212 [quant-ph] (2023).

30. Y. Hochberg, I. Charaev, S.-W. Nam, V. Verma, M. Colangelo, K. K. Berggren, Detecting sub-GeV dark matter with superconducting nanowires. *Phys. Rev. Lett.* **123**, 151802 (2019).

31. J. Chiles, I. Charaev, R. Lasenby, M. Baryakhtar, J. Huang, A. Roshko, G. Burton, M. Colangelo, K. Van Tilburg, A. Arvanitaki, S. W. Nam, K. K. Berggren, New constraints on dark photon dark matter with superconducting nanowire detectors in an optical haloscope *Phys. Rev. Lett.* **128**, 231802 (2022).

32. J. R. Clem, K. K. Berggren, Geometry-dependent critical currents in superconducting nanocircuits. *Phys. Rev. B.* **84**, 174510 (2011).

33. M. Jönsson, R. Vedin, S. Gyger, J. A. Sutton, S. Steinhauer, V. Zwiller, M. Wallin, J. Lidmar, Current crowding in nanoscale superconductors within the Ginzburg-Landau model. *Phys. Rev. Appl.* **17**, 064046 (2022).

34. M. K. Akhlaghi, H. Atikian, A. Eftekharian, M. Loncar, A. H. Majedi, Reduced dark counts in optimized geometries for superconducting nanowire single photon detectors. *Opt. Express* **20**, 23610–23616 (2012).

35. D. Henrich, L. Rehm, S. Dorner, M. Hofherr, K. Il'in, A. Semenov, M. Siegel, Detection efficiency of a spiral-nanowire superconducting single-photon detector. *IEEE Trans. Appl. Supercond.* **23**, 2200405–2200405 (2013).

36. I. Charaev, A. Semenov, S. Doerner, G. Gomard, K. Ilin, M. Siegel, Current dependence of the hot-spot response spectrum of superconducting single-photon detectors with different layouts. *Supercond. Sci. Technol.* **30**, 025016 (2017).

37. R. Baghdadi, E. Schmidt, S. Jahani, I. Charaev, M. G. W. Müller, M. Colangelo, D. Zhu, K. Ilin, A. D. Semenov, Z. Jacob, M. Siegel, K. K. Berggren, Enhancing the performance of superconducting nanowire-based detectors with high-filling factor by using variable thickness. *Supercond. Sci. Technol.* **34**, 035010 (2021).

38. J.-M. Xiong, W.-J. Zhang, G.-Z. Xu, L.-X. You, X.-Y. Zhang, L. Zhang, C.-J. Zhang, D.-H. Fan, Y.-Z. Wang, H. Li, Z. Wang, Reducing current crowding in meander superconducting strip single-photon detectors by thickening bends. *Supercond. Sci. Technol.* **35**, 055015 (2022).

39. D. Henrich, P. Reichensperger, M. Hofherr, J. M. Meckbach, K. Il'in, M. Siegel, A. Semenov, A. Zotova, D. Y. Vodolazov, Geometry-induced reduction of the critical current in superconducting nanowires. *Phys. Rev. B.* **86**, 144504 (2012).

40. H. L. Hortensius, E. F. C. Driessen, T. M. Klapwijk, K. K. Berggren, J. R. Clem, Critical-current reduction in thin superconducting wires due to current crowding. *Appl. Phys. Lett.* **100**, 182602 (2012).

41. S. Frasca, B. Korzh, M. Colangelo, D. Zhu, A. E. Lita, J. P. Allmaras, E. E. Wollman, V. B. Verma, A. E. Dane, E. Ramirez, A. D. Beyer, S. W. Nam, A. G. Kozorezov, M. D. Shaw, K. K. Berggren, Determining the depairing current in superconducting nanowire single-photon detectors. *Phys. Rev. B.* **100**, 054520 (2019).

42. A. Semenov, I. Charaev, R. Lusche, K. Ilin, M. Siegel, H.-W. Hübers, N. Bralović, K. Dopf, D. Y. Vodolazov, Asymmetry in the effect of magnetic field on photon detection and dark counts in bended nanostrips. *Phys. Rev. B.* **92**, 174518 (2015).

43. L. Zhang, L. You, D. Liu, W. Zhang, L. Zhang, X. Liu, J. Wu, Y. He, C. Lv, Z. Wang, X. Xie, Characterization of superconducting nanowire single-photon detector with artificial constrictions. *AIP Adv.* **4**, 067114 (2014).

44. X. Zhang, X. Zhang, J. Huang, C. Yang, L. You, X. Liu, P. Hu, Y. Xiao, W. Zhang, Y. Wang, L. Li, Z. Wang, H. Li, Geometric origin of intrinsic dark counts in superconducting nanowire single-photon detectors. *Superconductivity* **1**, 100006 (2022).

45. W. Zhang, Q. Jia, L. You, X. Ou, H. Huang, L. Zhang, H. Li, Z. Wang, X. Xie, Saturating intrinsic detection efficiency of superconducting nanowire single-photon detectors via defect engineering. *Phys. Rev. Appl.* **12**, 044040 (2019).

46. S. Strohauer, F. Wietschorke, L. Zugliani, R. Flaschmann, C. Schmid, S. Grotowski, M. Müller, B. Jonas, M. Althammer, R. Gross, K. Müller, J. J. Finley, Site-selective enhancement of superconducting nanowire single-photon detectors via local helium ion irradiation. *Adv. Quantum Technol.* **6**, 2300139 (2023).

47. M. W. Brenner, D. Roy, N. Shah, A. Bezryadin, Dynamics of superconducting nanowires shunted with an external resistor. *Phys. Rev. B.* **85**, 224507 (2012).

48. I. Charaev, E. K. Batson, S. Cherednichenko, K. Reidy, V. Drakinskiy, Y. Yu, S. Lara-Avila, J. D. Thomsen, M. Colangelo, F. Incalza, K. Ilin, A. Schilling, K. K. Berggren, Single-photon detection using large-scale high-temperature MgB2 sensors at 20 K. *Nat. Commun.* **15**, 3973 (2024).

49. Y.-Z. Wang, W.-J. Zhang, X.-Y. Zhang, G.-Z. Xu, J.-M. Xiong, Z.-G. Chen, Y.-Y. Hong, X.-Y. Liu, P.-S. Yuan, L. Wu, Z. Wang, L.-X. You, free-space coupled, large-active-area superconducting microstrip single-photon detector for photon-counting time-of-flight imaging. *Appl. Optics* **63**, 3130 (2024).

50. F. I. Allen, A review of defect engineering, ion implantation, and nanofabrication using the helium ion microscope. *Beilstein J. Nanotechnol.* **12**, 633–664 (2021).

51. O. Kahl, S. Ferrari, V. Kovalyuk, G. N. Goltsman, A. Korneev, W. H. P. Pernice, Waveguide integrated superconducting single-photon detectors with high internal quantum efficiency at telecom wavelengths. *Sci. Rep.* **5**, 10941 (2015).

52. H. Shibata, K. Shimizu, H. Takesue, Y. Tokura, Superconducting nanowire single-photon detector with ultralow dark count rate using cold optical filters. *Appl. Phys. Express.* **6**, 072801 (2013).

53. A. J. Kerman, D. Rosenberg, R. J. Molnar, E. A. Dauler, Readout of superconducting nanowire single-photon detectors at high count rates. *J. Appl. Phys.* **113**, 144511 (2013).

54. R. Flaschmann, L. Zugliani, C. Schmid, S. Spedicato, S. Strohauer, F. Wietschorke, F. Flassig, J. J. Finley, K. Müller, The dependence of timing jitter of superconducting nanowire single-photon detectors on the multi-layer sample design and slew rate. *Nanoscale* **15**, 1086–1091 (2023).

55. Y. Meng, K. Zou, N. Hu, L. Xu, X. Lan, S. Steinhauer, S. Gyger, V. Zwiller, X. Hu, Fractal superconducting nanowires detect infrared single photons with 84% system detection efficiency, 1.02 polarization sensitivity, and 20.8 ps timing resolution. *ACS Photonics* **9**, 1547–1553 (2022).

56. L. Zhang, L. You, L. Ying, W. Peng, Z. Wang, Characterization of surface oxidation layers on ultrathin NbTiN films. *Phys. C: Supercond. Appl.* **545**, 1–4 (2018).

57. A. J. Kerman, E. A. Dauler, W. E. Keicher, J. K. Yang, K. K. Berggren, G. Gol’tsman, B. Voronov, Kinetic-inductance-limited reset time of superconducting nanowire photon counters. *Appl. Phys. Lett.* **88**, 2–5 (2006).
